# Ureteroinguinal herniation complicated by obstructive uropathy and pyelonephritis

**DOI:** 10.1016/j.radcr.2023.09.089

**Published:** 2023-10-28

**Authors:** Fergus O'Herlihy, Philip J Dempsey, Dora Gorman, Edward McDermott, Eoin C Kavanagh

**Affiliations:** Mater Misericordiae University Hospital, Eccles St, Dublin, Ireland

**Keywords:** Hernia, Inguinal, Pyelonephritis, Ureter

## Abstract

Herniation of the ureters into the inguinal canal is a rare but recognized phenomenon. It may be noted incidentally on cross-sectional imaging, or when it presents with complications such as obstruction or infection. It is important to highlight the finding when present, as surgical intervention will be required in the majority of cases. We present a case of a 91-year-old man who developed obstructive uropathy and pyelonephritis due to inguinal herniation of the ureter.

## Introduction

Unlike other structures within the abdomen, the ureters do not frequently herniate outside of their normal location in the retroperitoneum. Herniation is more commonly seen following renal transplant, following which the ureters reside in the peritoneal cavity. Native ureteric herniation is very unusual, though case reports exist dating back to 1880 [1]. The condition is not without consequence, as demonstrated in our case report of a 91-year-old man who developed obstruction and subsequent pyelonephritis following ureteric herniation into the inguinal canal.

## Case report

A 91-year-old man presented to the emergency department with a 2-day history of dysuria, rigors, vomiting, and right-sided abdominal pain. His background history included prostate cancer, emphysema, diverticulitis, peptic ulcer disease, and hypertension. In addition, he was awaiting an outpatient urology consultation due to the incidental finding of a right-sided ureteric inguinal hernia, of which he had previously been asymptomatic.

He was pyrexic on presentation and tender in the right iliac fossa and right flank. Routine biochemistry revealed a mild neutrophilia (neutrophils 9.8 × 10^9^/L, normal 2-8 × 10^9^/L) and a significantly elevated C-reactive protein of 289 mg/L (normal range <7). Notably, renal function was normal. Urinalysis revealed raised leucocytes and microscopic haematuria.

Chest X-ray demonstrated bibasal linear atelectasis but no consolidation.

**Fig. 1:**
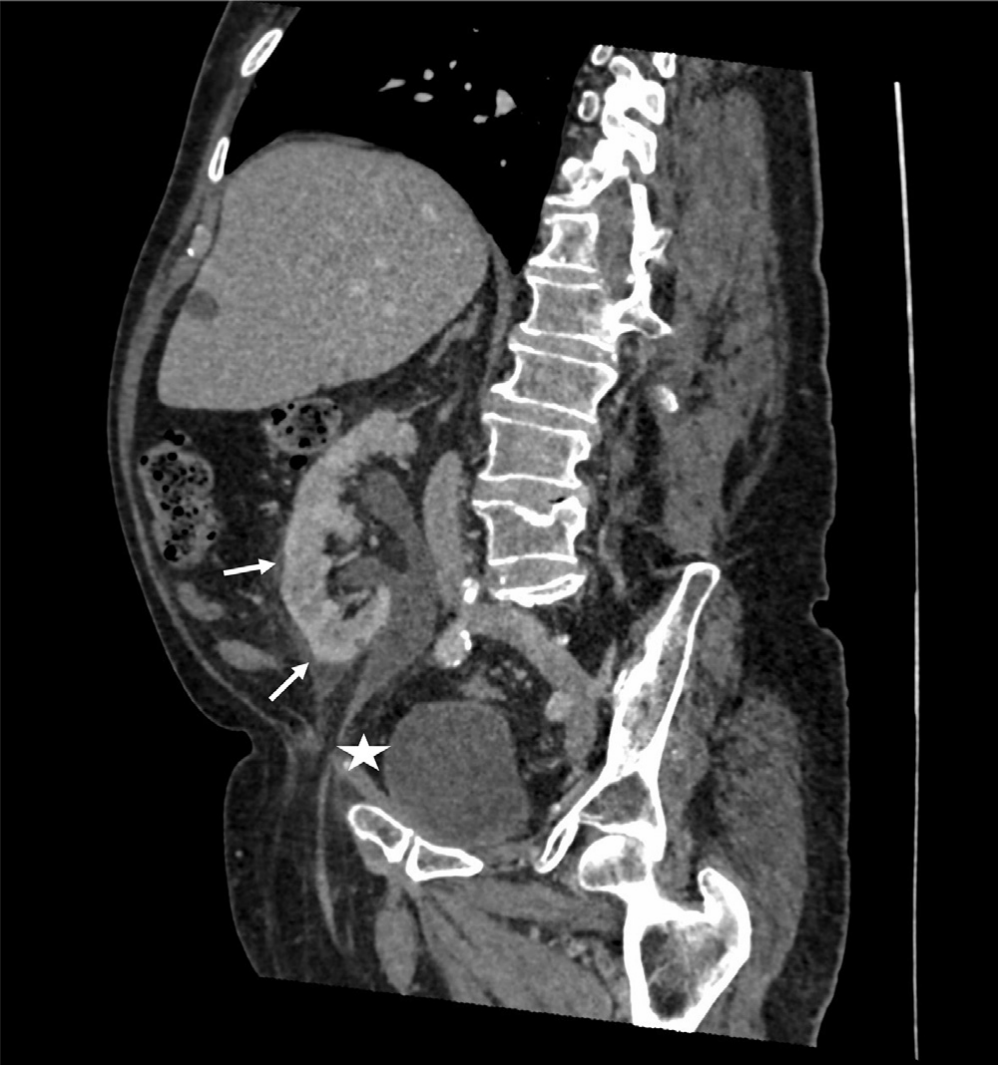
CT with coronal oblique reformat demonstrating hydronephrosis/hydroureter with a tapering point at the deep inguinal ring (white star). The kidney is hypoenhancing and there is perinephric fat stranding (white arrows).

**Fig. 2:**
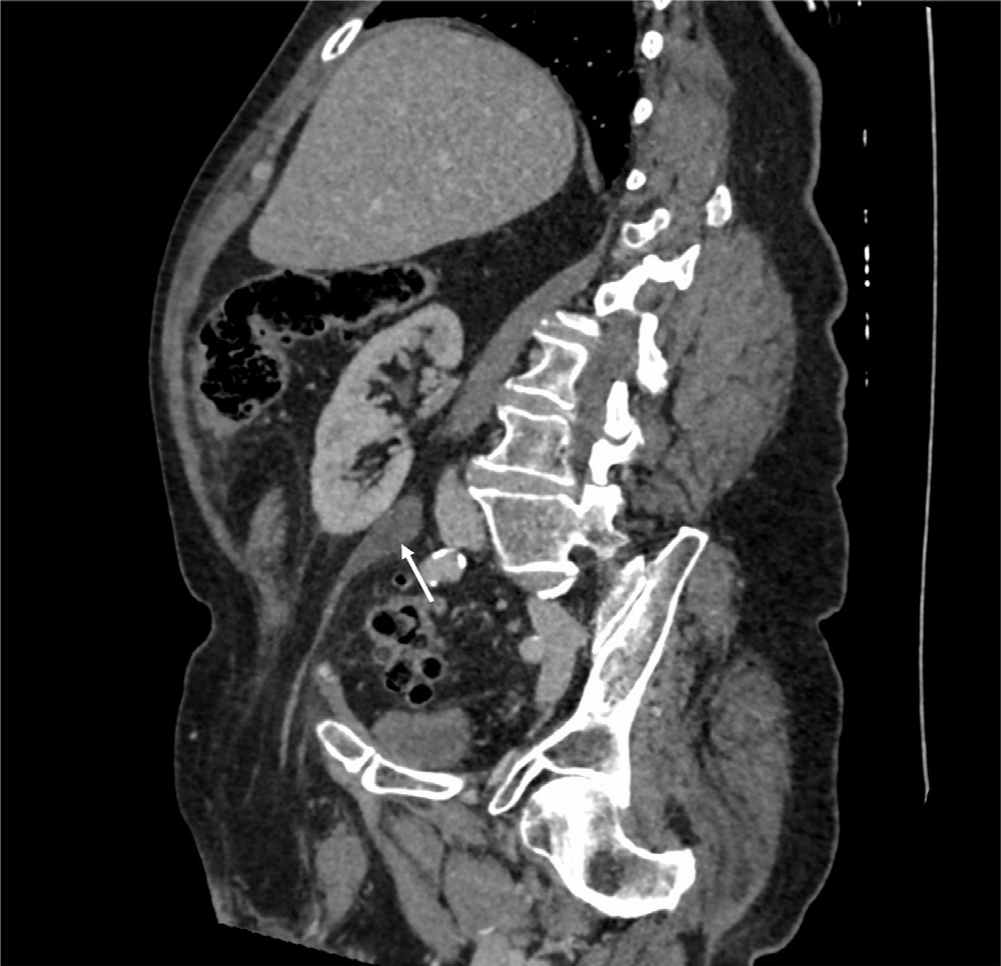
The patient's prior CT, showing milder hydronephrosis/hydroureter (white arrow) and an otherwise normal kidney.

**Fig. 3:**
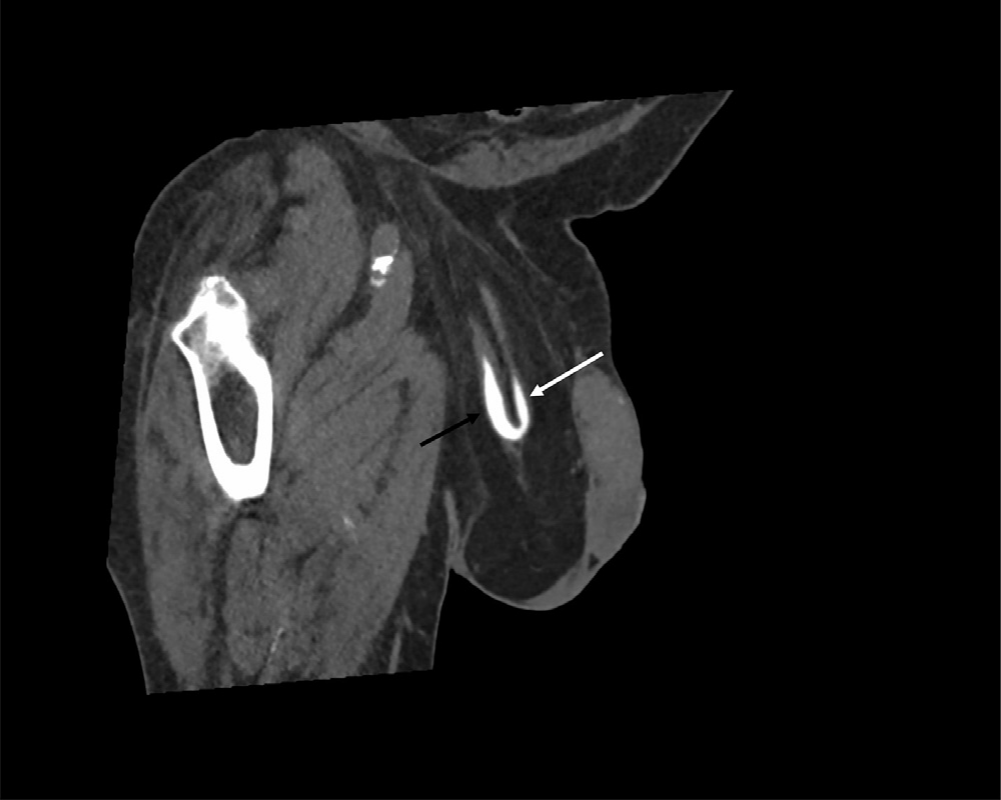
Delayed-phase CT with coronal oblique reformat. The inguinal hernia sac contains fat and the right ureter. The white arrow indicates the afferent loop, with the black arrow indicating the efferent loop.

Contrast-enhanced CT of the abdomen and pelvis was performed. This demonstrated an edematous right kidney with multifocal areas of cortical hypoenhancement, perinephric fat stranding and urothelial enhancement, consistent with acute pyelonephritis (Fig. 1). There was evidence of moderate hydronephrosis and hydroureter, which had progressed in the six months since the patient's previous CT (Fig. 2). The dilated ureter descended into the right inguinal canal (Fig. 3), before looping back into the pelvis and entering the bladder. There was a point of tapering of the afferent loop of ureter at the deep inguinal ring (Fig. 1).

The patient was treated with a 14-day course of intravenous antibiotics and experienced a significant clinical improvement. Following urology consultation the decision was made to pursue conservative management of the inguinal hernia, considering the patient's age and multiple comorbidities. Furthermore, it was noted that the patient had had just one prior episode of urinary tract infection 20 years prior, which did not require hospitalization. Percutaneous nephrostomy insertion or operative hernia repair was not required. He was discharged to the community and remained well at 6-week clinic review.

## Discussion

Ureteric herniation is an uncommon but recognized phenomenon, with reports of inguinal, femoral, sciatic, and intrathoracic herniation [1,2]. The first autopsy report of an inguinal ureteric herniation dates back to 1880, and the diagnosis was rarely made pre-operatively until the 21st century [1]. Approximately 140 cases have been reported in the literature, many of these occurring following renal transplant [3], [4], [5]–6]. Native ureteric herniation appears to be rarer still.

Inguinal ureteric hernias can be classified anatomically according to the presence or absence of a peritoneal hernia sac. Eighty percent of these hernias are paraperitoneal (containing a peritoneal sac) and are thought to occur when the ureter is drawn down into the scrotum due to its adherence to the posterior wall of the sac. These hernias are predominantly seen in men beyond the fourth decade of life and are more frequently right-sided, as in our patient [7].

The remaining 20% are extraperitoneal and have a more variable age at presentation, often occurring alongside other congenital abnormalities of the urinary tract [7,8]. Previously assumed to be a congenital hernia due to abnormal development of the ureter from the Wolffian duct, some authors have suggested that these hernias may in fact be acquired in nature [1]. Both paraperitoneal and extraperitoneal ureteric hernias are usually indirect inguinal hernias [7,9], though it is possible for a large direct inguinal hernia to involve both the bladder and ureter [1].

While there are prior reports of obstructive uropathy due to ureteric compression in the inguinal canal [3,^10^–12], it has to date been assumed that ureteric strangulation and obstruction is rare [7]. When obstruction does occur, it can be assumed that there will be an increased risk of superimposed pyelonephritis, as with other causes of obstructive uropathy [13]. We report an unusual case of a pre-existing inguinal ureteric hernia that developed obstruction and superimposed pyelonephritis. We have identified just 2 cases in the literature of urosepsis secondary to native ureteroinguinal hernia-related obstruction [11,14].

Our patient did not undergo operative repair of the hernia due to his multiple comorbidities and improvement with antibiotics. Furthermore, he had previously experienced only one case of urinary tract infection. The most frequently advocated treatment involves dissection and replacement of the ureter in the retroperitoneum followed by standard hernia repair, though in some cases ureteric resection with reanastomosis may be required [7]. In cases of obstruction, attempts at ureteric stent insertion (antegrade or retrograde) can be challenging due to the tortuosity of the ureteric loop in the inguinal canal [10]. Nephrostomy may be required as a temporary measure. Previous authors have advocated treatment in all cases, while acknowledging that there are cases to suggest that the condition can be well tolerated in some [1,^7^,8].

## Conclusion

Inguinal ureteric herniation is a rare phenomenon, particularly in native ureters. Serious complications including obstructive uropathy and superimposed pyelonephritis can occur. As such, the presence of an inguinal uretic hernia should be reported as clinically relevant even in the absence of obstruction.

## Patient consent

Written informed consent for publication of this case report has been obtained from the patient. The original informed consent form was retained for our records.
